# A RF Redundant TSV Interconnection for High Resistance Si Interposer

**DOI:** 10.3390/mi12020169

**Published:** 2021-02-08

**Authors:** Mengcheng Wang, Shenglin Ma, Yufeng Jin, Wei Wang, Jing Chen, Liulin Hu, Shuwei He

**Affiliations:** 1 Department of Mechanical & Electrical Engineering, Xiamen University, Xiamen 361102, China; 19920181152234@stu.xmu.edu.cn; 2 School of Electronic and Computer Engineering, Shenzhen Graduate School of Peking University, Shenzhen 518055, China; yfjin@pku.edu.cn; 3 National Key Laboratory of Science and Technology on Micro/Nano Fabrication, School of Electronic Engineering and Computer Science, Peking University, Beijing 100871, China; w.wang@pku.edu.cn (W.W.); j.chen@pku.edu.cn (J.C.); 4 Chengdu Ganide Technology Co., Ltd, Chengdu 610000, China; huliul@sina.com (L.H.); heshuwei_3@163.com (S.H.)

**Keywords:** millimeter-wave, redundant TSV, equivalent circuit model, S-parameters extraction

## Abstract

Through Silicon Via (TSV) technology is capable meeting effective, compact, high density, high integration, and high-performance requirements. In high-frequency applications, with the rapid development of 5G and millimeter-wave radar, the TSV interposer will become a competitive choice for radio frequency system-in-package (RF SIP) substrates. This paper presents a redundant TSV interconnect design for high resistivity Si interposers for millimeter-wave applications. To verify its feasibility, a set of test structures capable of working at millimeter waves are designed, which are composed of three pieces of CPW (coplanar waveguide) lines connected by single TSV, dual redundant TSV, and quad redundant TSV interconnects. First, HFSS software is used for modeling and simulation, then, a modified equivalent circuit model is established to analysis the effect of the redundant TSVs on the high-frequency transmission performance to solidify the HFSS based simulation. At the same time, a failure simulation was carried out and results prove that redundant TSV can still work normally at 44 GHz frequency when failure occurs. Using the developed TSV process, the sample is then fabricated and tested. Using L-2L de-embedding method to extract S-parameters of the TSV interconnection. The insertion loss of dual and quad redundant TSVs are 0.19 dB and 0.46 dB at 40 GHz, respectively.

## 1. Introduction

With the development of 5G communication technology and millimeter-wave radar, system-in-package (SIP) for high-frequency devices has become a popular research subject in industrial and academic fields. This is due to the technical benefits of small size, light weight, high integration, high density, and system performance improvement [1]. Traditionally, radio frequency (RF) SIP solution-based microwave printed circuit boards or high-performance ceramic substrates, such as HTCC (high temperature co-fired ceramics) and LTCC (low temperature co-fired ceramics), have faced challenges in terms of their precision of critical dimension and minimum size of redistribution lines and pitch. Due to the precise wiring capacity and the low mismach in material coefficient of thermal expansion (CTE), research works have been done to explore the feasibility as well as the technical advantage of TSV technology for RF application [2,3,4,5,6]. It has been found that the RF property of TSV becomes the key issue in this field as the natural property of Si as semiconductor, which is characterized in term of S-parameters. S-parameters are network parameters based on the relationship between incident wave and reflected wave. S_11_ named the return loss represents the reflection coefficient of incident port while S_21_ named insertion loss represents the transmission coefficient from incident port to the destination. To improve the S_21_ of TSV interconnection, optimization methods in materials, structural, and process flow are proposed. For example [7,8] using a high-resistance silicon substrate, the measured insertion loss of a single TSV is 0.35 dB at 20 GHz. References [9,10] designed the coaxial TSV structure containing two layers of conductors, the measured S_21_ of a TSV is −0.48 dB at 10 GHz. By optimizing the important electroplating process in the TSV manufacturing process [11,12,13], TSV can achieve bottom-up Cu filling, and a single TSV will have low DC resistance of 36.7 mΩ to ensure low RF loss. To our knowledge, the best test result is demonstrated by [14], which has an insertion loss of 0.53 dB at 75 GHz for a single TSV.

Various types of defects have been found in the manufacturing process of TSV. These include discontinuities and voids in the metal inside TSV caused by a poor sputtering seed layer or plating failure [15], pinholes and cracks of TSV oxidation caused by impurities in the insulating materials or deposition methods. The discontinuity of metal in the hole causes the signal channel to open and reflect most of the transmitted signal. Pinholes in the insulator around the TSV will cause a leakage current between the TSV and the substrate, resulting in a resistive short circuit [16]. Voids will cause the resistance of the interconnect to change, resulting in increased signal loss. These defects affect the signal transmission from the input to the receiver in different ways.

To address TSV failure, designs for a redundant TSV have been proposed. Samsung proposed a TSV redundant architecture with a switching method for 3D DDR3 DRAM (Samsung, Seoul, Korea) products [17]. Hsieh proposed a method for repairing shifted TSV [18]. Reference [19] proposed a redundant architecture based on routers. The current research on redundant TSVs is mainly focused on the logic 3D IC (integrated circuit) application, while there is little research regarding TSV’s RF application [20,21]. In this field, dense TSVs are not required for RF transmission which is favorable for redundant design. However, unlike the redundant design of TSV in logic IC, the participation of redundant TSV may change the characteristic impedance of RF TSV, and finally cause the degradation in RF insertion loss or electromagnetic compatibility issue. Therefore, special attention should be paid to RF redundant TSV design to guarantee that it can maintain an equivalent RF property with single RF TSV design whatever a defect occurs, which is the key point.

Therefore, a dual redundant and four redundant TSV interconnection designs are proposed for a high-resistivity Si interposer in this paper. The high-frequency performance is analyzed using a 3D field solver and a modified equivalent circuit model and compared with a single TSV interconnection. S-parameters are simulated when the redundant TSV has a via failure. Based on the proposed redundant TSV design, through the typical TSV process, samples are manufactured and tested. S-parameters of TSV interconnection are obtained by de-embedding. In view of the process factors that cause the measured radio frequency performance to decline, analysis and optimization simulation are carried out and an agreement is obtained. Finally, the high-frequency performance of the redundant TSV structure proposed in this paper is compared with the published single TSV to show its technological advantage.

## 2. Structural Design

**Figure 1:**
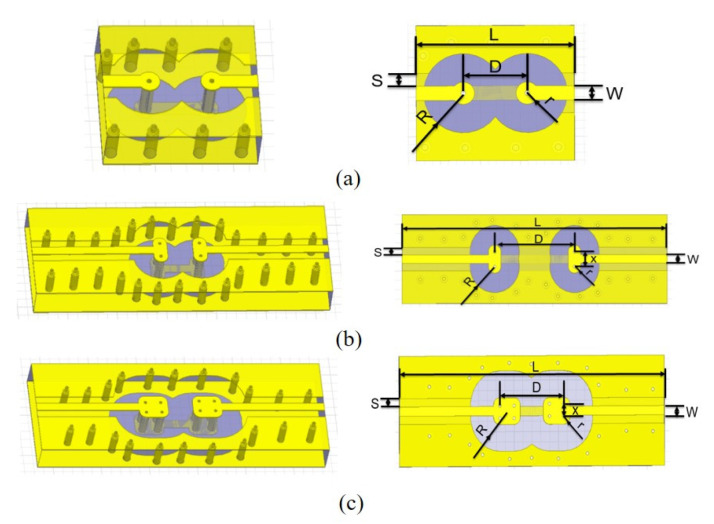
Test structure HFSS model: (**a**) single TSV; (**b**) dual redundant TSV; (**c**) quad redundant TSV.

Figure 1 shows the proposed redundant RF TSV. Figure 1b,c are dual and quad redundant RF TSVs on high resistivity Si substrate, respectively, while Figure 1a is a traditional single RF TSV as a reference. For ease of use in further test and S-parameters extraction, RF TSVs on high resistivity Si substrate are connected by coplanar waveguide (CPW) lines, which have a designed impedance of 50 Ω and dimensions are summarized in Table 1.

**Figure 2:**
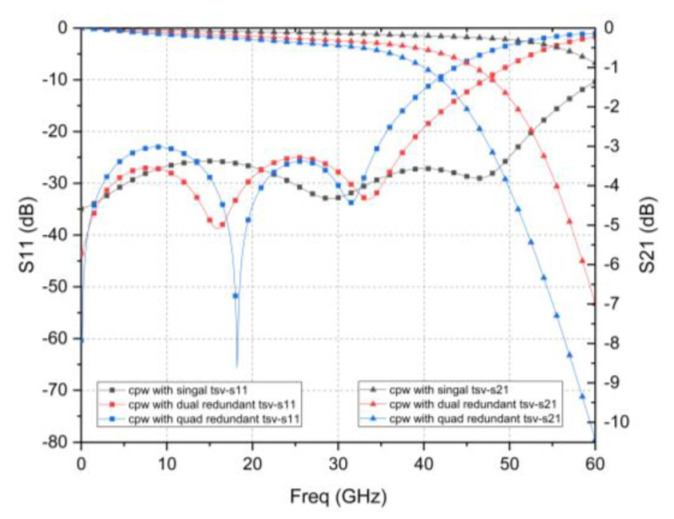
Test structure simulation result.

Figure 2 shows simulated S-parameters with HFSS model. It can be seen that at a frequency of 40 GHz, the insertion losses of a single TSV interconnect test structure, dual redundant TSV interconnect test structure, and quad redundant TSV interconnect test structure design are 0.197 dB, 0.538 dB, and 0.998 dB, respectively. The S_11_ parameter is less than −15 dB. The results show that it has a good high-frequency transmission performance. At the same time, as the number of redundant TSVs increases, the S_21_ parameter shows that the insertion loss value gradually degrades, and the S_11_ parameter shows that the resonance frequency gradually decreases.

**Figure 3:**
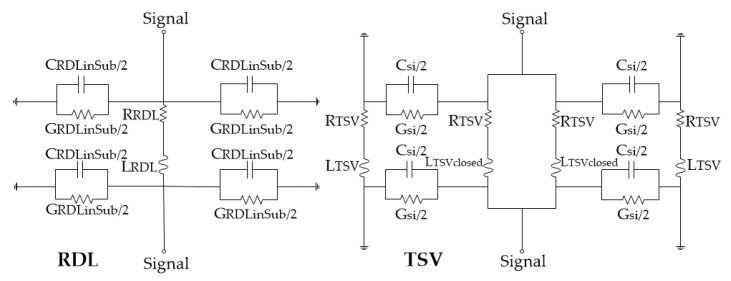
Equivalent circuit models of dual redundant TSV test structure.

To better understand the effect of the redundant TSVs on the high-frequency transmission performance, a modified lumped circuit model is established for the dual redundant TSV test structure in this paper, as shown in Figure 3. The parameters of the symbols in the model are listed in Table 2. The values of each lumped element can be calculated by applying the dimensions [22,23,24,25] and material properties [26,27,28] in the following equations. The diameter, width, length, thickness, height, and spacing is symbolized d, w, l, t, h, and p, respectively.

(1) $$\delta_{Cu}=\frac{1}{\sqrt{\pi f\mu_{0}\sigma_{Cu}}}$$

(2) $$R_{RDL\_DC}=\frac{l_{RDL}}{\sigma_{Cu}w_{RDL}t_{RDL}}$$

(3) $$R_{RDL\_AC}=\frac{l_{RDL}}{\sigma_{Cu}w_{RDL}\delta_{\begin{array}{l} Cu \end{array}}}$$

(4) $$R_{RDL}=\sqrt{R_{RDL\_DC}{}^{2}+R_{RDL\_AC}{}^{2}}$$

(5) $$L_{RDL}=\frac{\mu_{0}l_{RDL}}{2\pi }\left(\ln\left(\frac{2l_{RDL}}{w_{RDL}+t_{RDL}}\right)+\frac{1}{2}+\frac{w_{RDL}+t_{RDL}}{{3l}_{RDL}}\right)$$

(6) $$C_{RDLinSub}=\frac{\varepsilon_{0}\varepsilon_{si}l_{RDL}w_{RDL}}{h_{si}}$$

(7) $$G_{RDLinSub}=\frac{\sigma_{si}l_{RDL}w_{RDL}}{h_{si}}$$

(8) $$R_{TSV\_DC}=\frac{h_{\mathrm{TSV}}}{\sigma_{Cu}\pi {\left(d_{TSV}/2\right)}^{2}}$$

(9) $$R_{TSV\_AC}=\frac{h_{\mathrm{TSV}}}{\sigma_{Cu}\pi ({\left(d_{TSV}/2\right)}^{2}-{\left(d_{TSV}/2-\delta_{Cu}\right)}^{2})}$$

(10) $$R_{TSV}=\sqrt{R_{TSV\_DC}{}^{2}+R_{TSV\_AC}{}^{2}}$$

**Figure 4:**
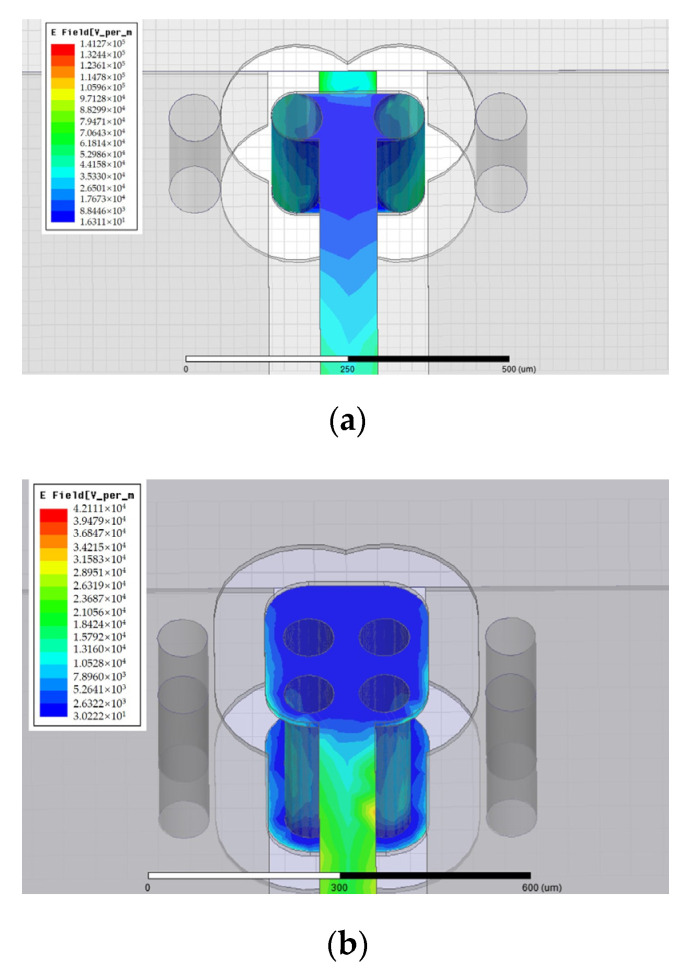
Electric field diagram: (**a**) dual redundant TSV; (**b**) quad redundant TSV.

When the alternating current in the same direction flows in the redundant TSV copper column, the alternating magnetic field generated by each current will generate eddy currents on adjacent TSVs, resulting in uneven current distribution in the TSV copper column and the proximity effect. The electric field distribution of the redundant TSV design proposed in this paper is shown in Figure 4. It is obvious that the internal electric field of TSV is concentrated in the edge area. Therefore, the equivalent resistance and inductance of TSV are changed, which needs to be considered when calculating the equivalent circuit.

(11) $$PF=\frac{p_{TSV}/d_{TSV}}{\sqrt{{\left(p_{TSV}/d_{TSV}\right)}^{2}-1}}$$

(12) $$R_{TSV_{closed}}=PF\cdot R_{TSV\_AC}$$

(13) $$L_{TSV}=\frac{\mu_{0}h_{TSV}}{2\pi }\left[\ln\left(\frac{h_{TSV}}{d_{TSV}/2}+\sqrt{{\left(\frac{h_{TSV}}{d_{TSV}/2}\right)}^{2}+1}\right)+\frac{d_{TSV}/2}{h_{TSV}}-\sqrt{{\left(\frac{d_{TSV}/2}{h_{TSV}}\right)}^{2}+1}\right]+\frac{R_{TSV\_AC}}{2\pi f}$$

(14) $$M_{TSV}=\frac{\mu_{0}h_{TSV}}{2\pi }\left[\ln\left(\frac{h_{TSV}}{p_{TSV}}+\sqrt{{\left(\frac{h_{TSV}}{p_{TSV}}\right)}^{2}+1}\right)+\frac{p_{TSV}}{h_{TSV}}-\sqrt{{\left(\frac{p_{TSV}}{h_{TSV}}\right)}^{2}+1}\right]$$

(15) $$L_{TSV_{closed}}=L_{TSV}+M_{TSV}$$

(16) $$Csub=\frac{\pi \varepsilon_{0}\varepsilon_{si}h_{TSV}}{cosh^{-1}\left(\frac{p_{TSV}}{d_{TSV}}\right)}$$

(17) $$Gsub=\frac{\pi \sigma_{si}h_{TSV}}{cosh^{-1}\left(\frac{p_{TSV}}{d_{TSV}}\right)}$$

**Figure 5:**
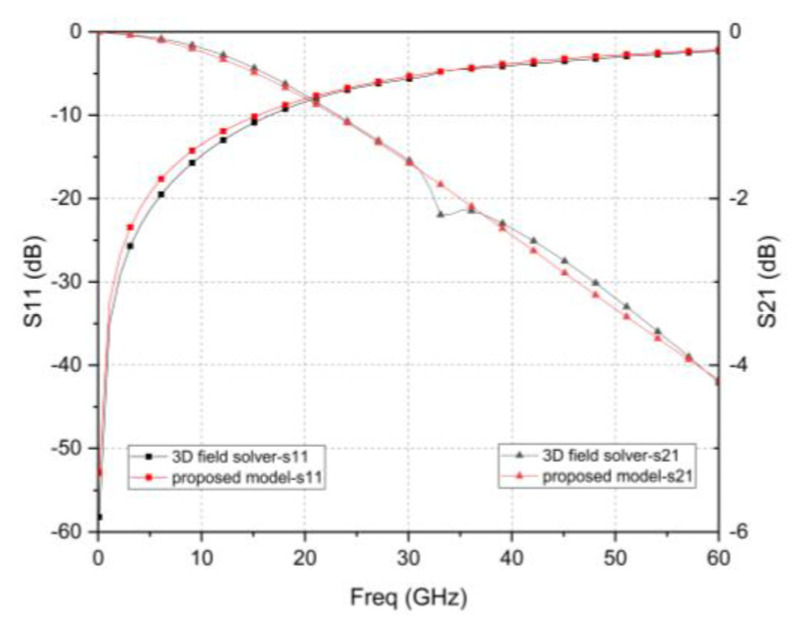
S-parameters results of dual redundant TSV structure obtained by 3D FEM solver and equivalent circuit model.

The simulation results with HFSS and the established equivalent circuit model in Figure 3 are shown in Figure 5. The similarity of the curvature of the amplitude curves of S_11_ and S_21_ indicates that the establishment of the equivalent circuit model is correct. It can be seen from the equivalent circuit model that, at high frequencies, the main factors that affect the S-parameters are inductance and resistance. As the redundant structure contains multiple RF TSVs, the proximity effect between TSVs will increase, and the overall inductance and resistance will increase due to the additional RDL required to connect multiple TSVs. Therefore, an increase in the number of redundant TSVs will cause the resonance frequency to appear in the lower frequency range, and the high-frequency loss will degrade significantly.

**Figure 6:**
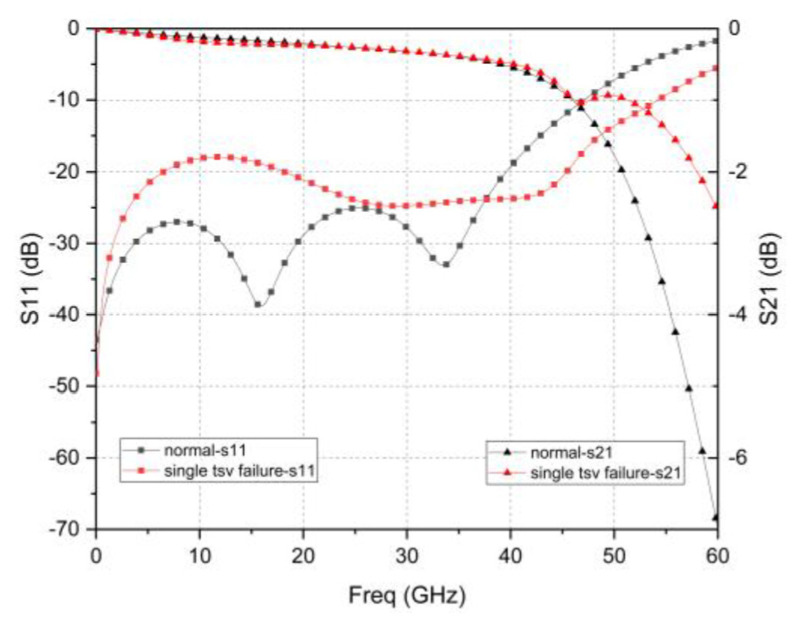
Dual redundant TSV failure simulation results.

**Figure 7:**
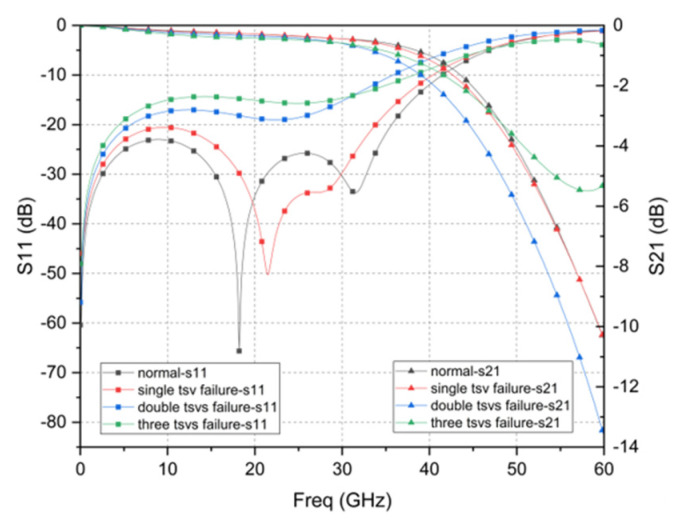
Quad redundant TSV failure simulation results.

In order to verify the feasibility of the RF redundant TSV scheme, Figure 6 and Figure 7 shows that the S-parameters of dual and quad redundant RF TSV test structure when failure occurs. It can be found that it has a higher resonance frequency and smaller insertion loss as the number of failed TSV increases. This result is basically consistent with the above analysis. Furthermore, it can achieve reliable function at 0–44 GHz regardless of what failure occurs.

## 3. Fabrication

**Figure 8:**
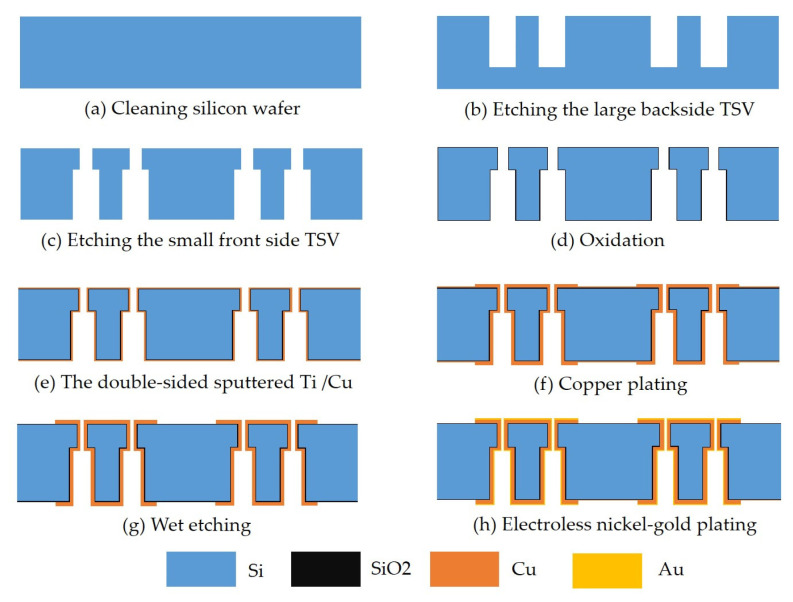
Process design.

**Figure 9:**
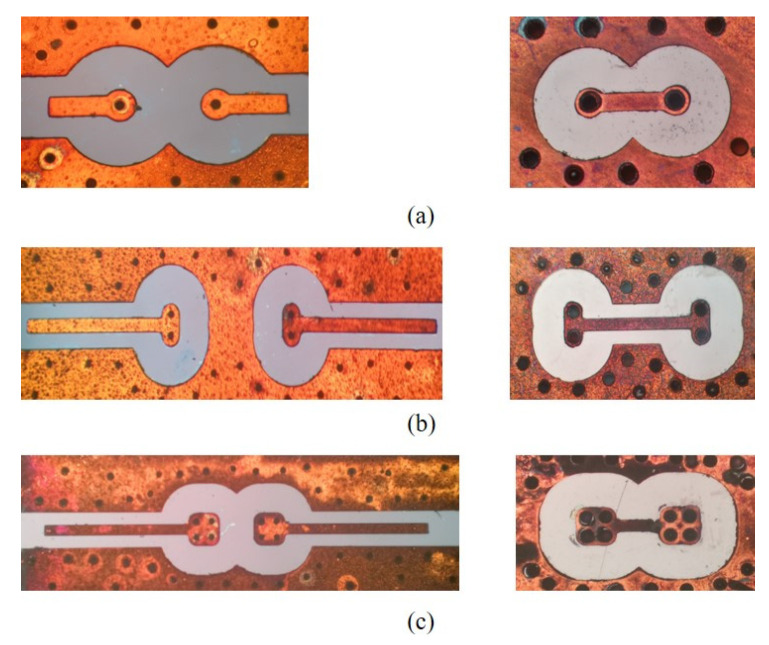
Test structure physical photo: (**a**) single TSV; (**b**) dual redundant TSV; (**c**) quad redundant TSV.

**Figure 10:**
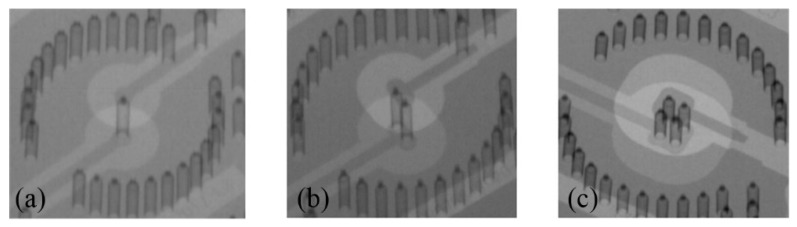
Test structure X-ray detection map: (**a**) single TSV; (**b**) dual redundant TSV; (**c**) quad redundant TSV.

Figure 8 shows the main steps for fabricating the redundant RF TSV sample. They include the following: (a) First, a 300 μm high-resistance silicon wafer is cleaned in acetone and isopropanol. (b) The fabrication of the large backside TSV is completed by photolithography and deep reactive ion etching. (c) The fabrication of the small front side TSV is completed by back engraving and deep reactive ion etching. (d) After standard cleaning, a high-temperature thermal oxygen process is used to form a dense 100 nm SiO2 insulating layer on the surface of the high resistance silicon wafer and the sidewall of the TSV. (e) The double-sided sputtered adhesion layer Ti and seed layer Cu is fabricated. (f) Double-sided lithography and thickening of the surface local copper layer and TSV hole copper layer is conducted by electroplating in a copper sulfate solution. (g) Copper plating area mask protection is carried out. Removing the excess Cu seed layer and Ti adhesion layer by wet etching. (h) Electroless nickel-gold plating is con-ducted on the Cu layer. Figure 9 provides a physical diagram of the completed production. As shown in Figure 10, the metal filling in the TSV hole is good under X-ray detection. To extract the S-parameters of the TSV interconnect structure, a transmission line is also manufactured at the same wafer.

## 4. RF Test and Analysis

The redundant RF TSV samples were tested using a GSG probe in a semi-automatic probe station, which was connected with an AV3629 high-performance microwave integrated vector network analyzer. Before the test, the measurement system was firstly calibrated using the classic SOLT calibration method, including short circuit, open circuit, load, and straight through four standard structures, to correct the system error, stripping probe and cable parasitic parameters [29]. The measured insertion losses at 40 GHz for a single TSV interconnect test structure, dual redundant TSV interconnect test structure, and quad redundant TSV interconnect test structure are 0.721 dB, 1.18 dB, and 1.635 dB, respectively.

**Figure 11:**
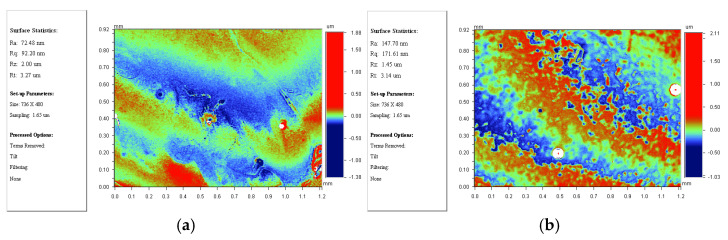
Surface roughness test results: (**a**) small roughness area; (**b**)large roughness area.

**Figure 12:**
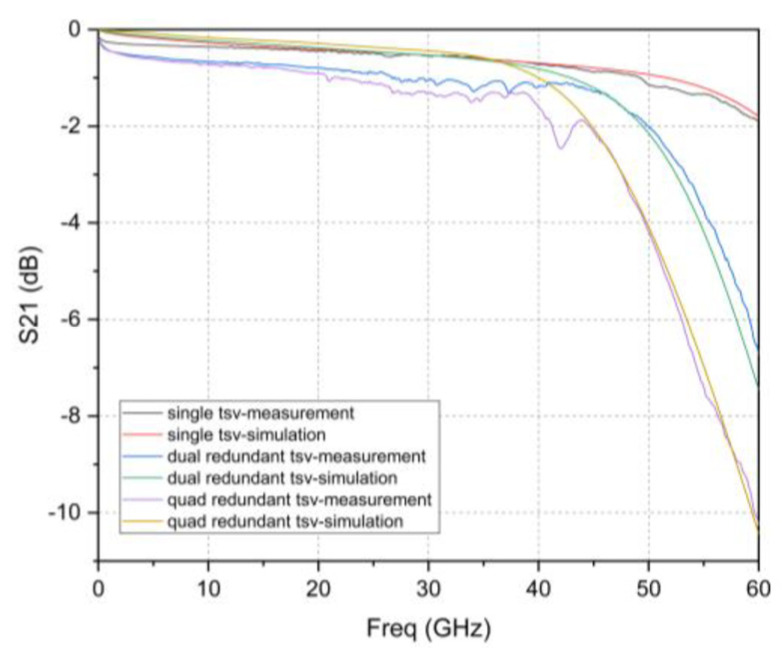
Test structure S_21_ parameter measurement results and optimized simulation results of HFSS.

Compared with the simulation results in Section 2, the maximum deviations of the insertion loss of the simulation and testing of a single TSV, dual redundant TSV and four redundant TSV test structures are 0.53 dB, 0.84 dB, and 0.95 dB in the range of 0–40 GHz, which may be caused by the use of the ideal Cu layer in the simulation. However, the fabricated Cu layer has some differences with ideal Cu layer, such as surface roughness and resistivity. Figure 11 is a captured photo of the Cu layer by a profiler during the process, which shows the roughness is approximately 60–70 nm and some local regions reach about 150 nm due to oxidation. Table 3 summarizes the tested resistivity, which has an average resistivity of 12.79 μΩ·cm. Because surface roughness of the copper generates parasitic inductance, the surface impedance will change and results in conductor loss [30]. Especially when the skin depth corresponding to the operating frequency is less than or equal to the surface roughness, the effect of surface roughness will become very significant [31,32]. Additionally, the resistivity of the conductor also affects the conductor loss. To testify this point, using the monitored data, the simulation is optimized and repeated in HFSS model, and the results are compared with the test results shown in Figure 12. It can be seen that the deviation is relatively reduced, and the higher the frequency, the better the fit. This proves that the roughness and resistivity of the conductor have an impact on high-frequency performance. It can also be seen in the figure that the gap between the measured and simulated results of the four-redundancy is significantly larger compared to a single TSV. Since the resistivity and roughness of the conductor in the hole cannot be measured, the parameters of the conductor on the plane can only be used instead. As the number of RF TSV holes increases, the error accumulation is greater.

## 5. S-Parameter Extraction

**Figure 13:**
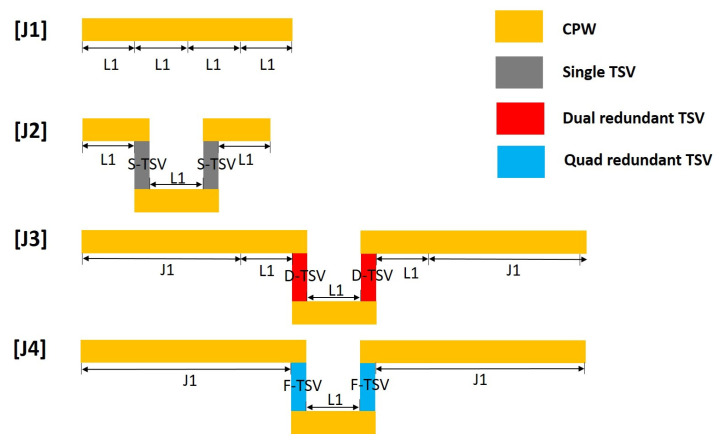
Schematic diagram of test structure de-embedding.

To obtain the precise value of insertion loss contributed by RF redundant TSVs, de-embedding was conducted. According to the relevant theory of microwave network parameters, conversion into ABCD parameters with cascade characteristics for RF redundant TSV sample structures was carried out via parameter transformation and matrix operation [33]. The 1000 μm CPW test structure is viewed as four 250 μm CPW connections. A single RF TSV interconnect and redundant RF TSV interconnect test structure is viewed as three CPW and two TSV interconnect structures, as shown in Figure 13. To simplify the description, J1, J2, J3, and J4 are used to represent the CPW, single TSV interconnect, dual redundant TSV interconnect, and quad redundant TSV interconnect test structure. L1 represents 250 μm CPW and S-TSV represents a single TSV mutual connected structure, D-TSV represents a dual redundant TSV interconnect structure, and Q-TSV represents a quad redundant TSV interconnect structure.

The ABCD parameters corresponding to the four unit structures are represented by square brackets “[]” and the tag name. The ABCD parameters of the unit structure are multiplied by the unit structure to represent the ABCD parameters of the three test structures J1, J2, J3, and J4 as

(18) [J1]=[L1][L1][L1][L1]

(19) [J2]=[L1][S−TSV][L1][S−TSV][L1]

(20) [J3]=[J1][L1][D−TSV][L1][D−TSV][L1][J1]

(21) [J4]=[J1][Q−TSV][L1][Q−TSV][J1]

The number of frequency points of the high-frequency measurement is marked as N, and the size of the ABCD parameters matrix of all the above test structures and unit structures is 2 × 2 × N. In the calculation, an N-step loop is set to perform a 2 × 2 matrix operation. The de-embedding process solves a single TSV interconnect S-TSV, a dual redundant TSV interconnect D-TSV, and a quad-redundant TSV interconnect Q-TSV using the above four matrix equations. Through the operations of square root and inversion matrix, the ABCD parameters matrix of each unit structure is obtained as

(22) $$\left[L1\right]={\left[J1\right]}^{\frac{1}{4}}$$

(23) $$\left[S-TSV\right]={\left[L1\right]}^{-1}{\left(\left[J2\right]{\left[L1\right]}^{-1}\right)}^{\frac{1}{2}}$$

(24) $$\left[D-TSV\right]={\left[L1\right]}^{-1}{\left({\left[J1\right]}^{-1}\left[J3\right]{\left[J1\right]}^{-1}{\left[L1\right]}^{-1}\right)}^{\frac{1}{2}}$$

(25) [L2]=[L1][L1][L1]

(26) $$\left[Q-TSV\right]={\left[L1\right]}^{-1}{\left({\left[L2\right]}^{-1}\left[J4\right]{\left[J1\right]}^{-1}\right)}^{\frac{1}{2}}$$

where [L2] is an intermediate variable for simplifying expressions. According to the transformation relationship between ABCD parameters and S-parameters, the ABCD parameters of each unit structure is converted into S-parameters, and the transmission characteristics of the three TSV interconnections can be compared. Microwave network parameter conversion and other operations in the de-embedding process are performed in MATLAB software(MATLAB, R2020a, MathWorks, Natick, Mass, USA).

**Figure 14:**
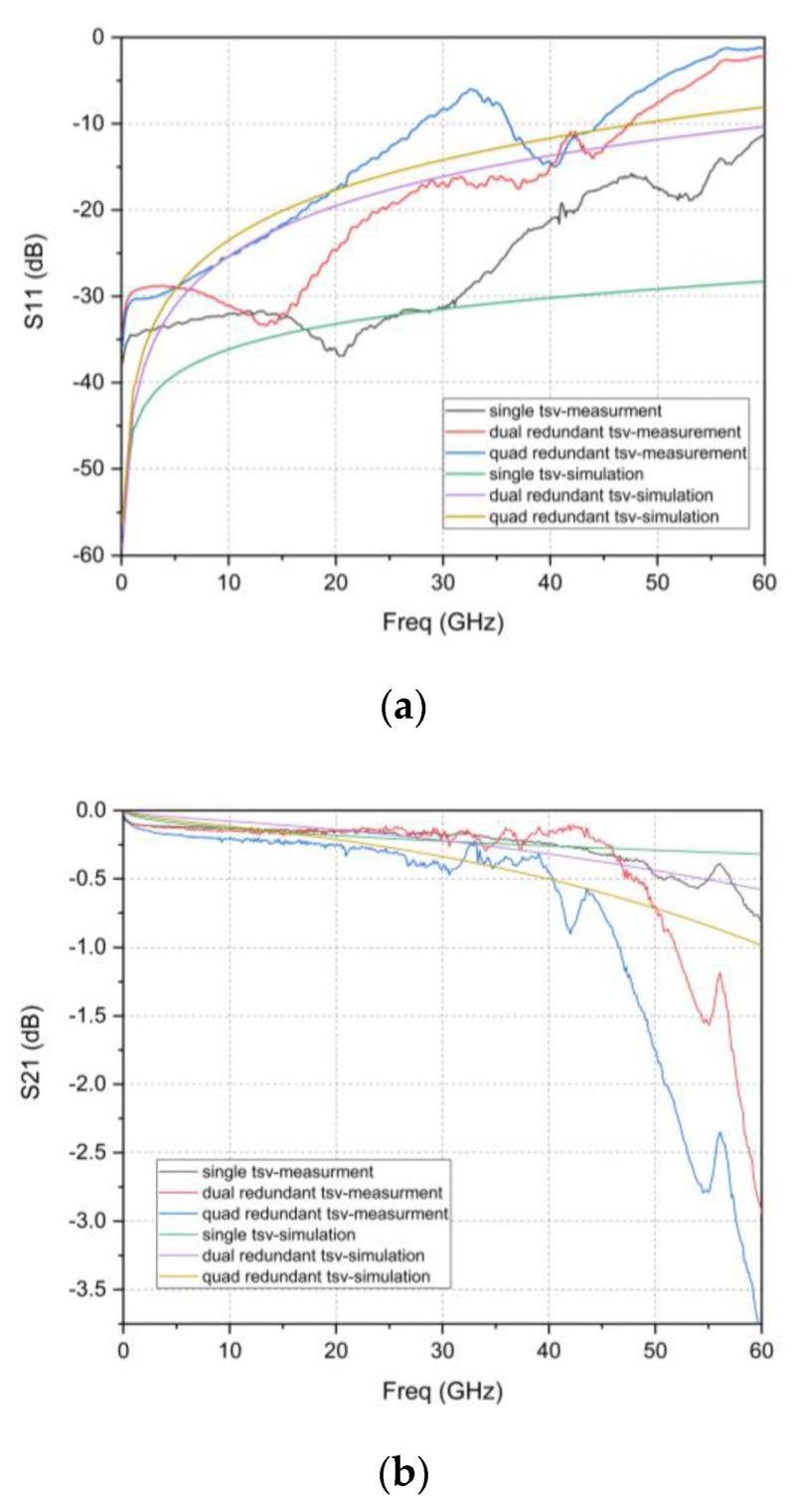
TSV interconnects S_21_ parameter measurement results and simulation results of the equivalent lumped components models: (**a**) S_11_; (**b**) S_21_.

Using the above de-embedding process, the S-parameters of the two kinds of RF TSV design are obtained, as shown in Figure 14. It can be observed that the S_21_ values of the three TSV interconnects are close when the frequency is less than 40 GHz, and the gap is in the range of 0.25 dB. When the frequency is greater than 40 GHz, the S_21_ value of the dual redundant and quad-redundant TSV interconnects degrade rapidly versus frequency, and the insertion loss of the quad-redundant TSV interconnect significantly increases. The test results of the TSV interconnection and the simulation results of the equivalent lumped components models are compared. The following points can be seen from Figure 14: (a) The simulated and measured insertion loss values gradually degrade as frequency increases. (b) Both simulation and measurement results show a similar trend that the insertion loss value degrades as the number of RF Redundant TSV increase. (c) The S-parameter curve of the TSV interconnection extracted from the RF measurement results shows some fluctuation, bringing out maximum deviation values of 0.07 dB, 0.17 dB, and 0.12 dB in the 0–40 GHz range between the simulation and measured results for a single TSV, dual redundant TSV, and four redundant TSV respectively. These fluctuations should be ascribed to the surface roughness or random discontinuities in the deposited Cur layer on the sidewall of TSV, of which, the later one is especially hard to discern or characterize to our knowledge. This is the reason why the current simulation based equivalent lumped component circuit model only considers the change of conductor resistivity as well. However, even with the deviations due to the fluctuations, those founding is sufficient to draw a conclusion that it has a competing RF property with single RF TSV for Redundant RF design, which is the most important to this research and highlighted in the Table 4 as well.

At present, there is no report of RF redundant TSV interconnection sample measurement result, although few papers proposed RF redundant RF TSV design. Table 4 compares redundant RF TSVs proposed in this paper with single TSV products presented in recent years. It can be seen from the table that when the frequency is less than 40 GHz, the RF Redundant TSV interconnection sample fabricated in this work is in the same level with traditional single RF TSV in term of RF insertion loss, but it has a better capacity to resist failure risk of RF TSVs.

## 6. Conclusions

This paper presented a RF redundant TSV design for a high resistance Si interposer as a package substrate. To verify the feasibility of the scheme, two test structures for connecting CPW transmission lines through redundant TSVs were designed to be able to work in 0–40 GHz. Modeling and simulation were carried out using HFSS, conclusion can be drawn from the obtained S-parameters that the more the number of RF redundant TSV, the lower the resonance frequency, and the greater the insertion loss. This was also solidified with the results obtained with the established modified equivalent circuit model for RF redundant TSV interconnection. The simulation also shows that the designed RF redundant TSV interconnection is capable to work in the range of 0–40 GHz without unacceptable RF property degradation when failure occurs. RF redundant TSV test vehicles were fabricated and tested, while an improved simulation factoring in nonideal factors such as surface roughness and resistivity is also taken. The result of the test shows an agreement with simulation. The tested insertion loss of the single TSV, dual redundant TSV and quad redundant TSV after de-embedding is 0.22, 0.19, and 0.46 dB at 40 GHz, respectively, which is close to the reported single TSV design. However, redundant TSV offers a better capacity to resist failure risk of TSV.

## Figures and Tables

**Table 1:** Parameters of the test structure (μm).

Table 1	L	S	W	D	R	r	x
Single TSV	1000	70	100	400	250	75	-
Dual redundant TSV	3000	70	100	400	250	75	160
Quad redundant TSV	3000	70	100	640	250	75	120

**Table 2:** Symbols and parameters in the equivalent circuit model for TSV and RDL.

Symbol	Parameter
R_RDL_/R_TSV_	Resistance of RDL/TSV
L_RDL_	Self-Inductance of RDL
L_TSVclosed_	Inductance of RDL under the influence of proximity effect
C_RDLinSub_/C_sub_	Capacitance between RDL/TSV and substrate
G_RDLinSub_/G_sub_	Conductance between RDLs/TSVs in silicon substrate
PF	Proximity effect correction factor
d_Cu_	Skin depth

**Table 3:** Resistivity test results.

Points	Resistivity/μΩ·cm
1	4.56
2	12.44
3	13.95
4	12.43
5	18.24
6	12.36
Average value	12.79

**Table 4:** Comparison RF TSV for high frequency applications.

Ref.	Substrate Material	Type of Vias	Transmission Loss of One Transition (dB)		Via Size (μm)	Via Length (μm)
			10 GHz	40 GHz		
[34]	Glass	Single TGV	0.03	0.22	Φ55	366
[35]	LCP	Single Via	0.071	0.12	Φ55	51
[36]	Si(HR)	Single TSV	0.05	—	Φ100	300
[37]	Si(HR)	Single TSV	0.04	—	Φ8 & Φ90	25 & 280
[38]	Si(HR)	Single TSV	1.6	—	Φ40	120
[39]	Si(HR)	Single TSV	0.37	—	Φ10	100
This Work	Si(HR)	Single TSV	0.11	0.22	Φ40 & Φ80	50 & 250
		Dual redundant TSV	0.14	0.19		
		Quad redundant TSV	0.2	0.46		

## Data Availability

The data presented in this study are available on request from the corresponding author. The data are not publicly available due to the experimental data needs to be further researched in the future.
